# NGSremix: a software tool for estimating pairwise relatedness between admixed individuals from next-generation sequencing data

**DOI:** 10.1093/g3journal/jkab174

**Published:** 2021-05-20

**Authors:** Anne Krogh Nøhr, Kristian Hanghøj, Genís Garcia-Erill, Zilong Li, Ida Moltke, Anders Albrechtsen

**Affiliations:** 1 Department of Biology, The Bioinformatics Centre, University of Copenhagen, 2200 Copenhagen N, Denmark; 2 H. Lundbeck A/S, 2500 Valby, Denmark

**Keywords:** relatedness, genotype likelihoods, maximum likelihood estimation, admixture, low-depth NGS data

## Abstract

Estimation of relatedness between pairs of individuals is important in many genetic research areas. When estimating relatedness, it is important to account for admixture if this is present. However, the methods that can account for admixture are all based on genotype data as input, which is a problem for low-depth next-generation sequencing (NGS) data from which genotypes are called with high uncertainty. Here, we present a software tool, NGSremix, for maximum likelihood estimation of relatedness between pairs of admixed individuals from low-depth NGS data, which takes the uncertainty of the genotypes into account via genotype likelihoods. Using both simulated and real NGS data for admixed individuals with an average depth of 4x or below we show that our method works well and clearly outperforms all the commonly used state-of-the-art relatedness estimation methods PLINK, KING, relateAdmix, and ngsRelate that all perform quite poorly. Hence, NGSremix is a useful new tool for estimating relatedness in admixed populations from low-depth NGS data. NGSremix is implemented in C/C++ in a multi-threaded software and is freely available on Github https://github.com/KHanghoj/NGSremix.

## Introduction

Estimation of genetic relatedness between pairs of individuals is important in many genetic research areas. For example, genetically related individuals are removed or accounted for in genome-wide association studies to avoid an inflated false positive rate (Morris and Cardon 2019). The genetic relatedness between a pair of individuals is most often described using the concept of identity-by-descent (IBD), which is genetic identity due to recent common ancestry. To quantify the genetic relatedness between a pair of individuals the summary statistics $R=(k_{0},k_{1},k_{2})$ can be used, where $k_{0}$, $k_{1}$, and $k_{2}$ are the proportion of the genome where a pair of individuals share 0, 1, or 2 alleles IBD, respectively (Weir *et al.* 2006). These statistics are useful because their expected values differ between different types of familial relationships, with the expected value of $k_{0}$ being 1 for unrelated individuals and in general it is smaller the closer related two individuals are. For instance, the expected values of *R* for sibling pair is (0.25,0.5,0.25) and for a parent offspring pair, it is (0,1,0). *R* can therefore be used to quantify how closely related two individuals are. There are also other IBD-based summary statistics of genetic relatedness, like the kinship coefficient, however, we will here focus on *R*, because other such summary statistics can be calculated from it. For example, the kinship coefficient is simply $\frac{\frac{k_{1}}{2}+k_{2}}{2}$.

Several methods exist for estimation of genetic relatedness between a pair of individuals. When choosing a method to use it is important to consider what type of data is available, where the individuals are from and whether the individuals are admixed. Most current methods are based on the assumption that the individuals are all from a single homogeneous population (Thompson 1975; Ritland 1996; Milligan 2003; Purcell *et al.* 2007; Albrechtsen *et al.* 2009), including the commonly used method implemented in PLINK (Purcell *et al.* 2007). If this assumption is violated the estimation of relatedness will be biased and relationships can be miss-classified (Rohlfs *et al.* 2012; Thornton *et al.* 2012; Moltke and Albrechtsen 2014). To address this problem, several methods, such as PC-Relate (Conomos *et al.* 2016), REAP (Thornton *et al.* 2012), RelateAdmix (Moltke and Albrechtsen 2014), and KING (Manichaikul *et al.* 2010) have been developed. These methods are all developed to be applied to diallelic genotype data, like single nucleotide polymorphism (SNP) chip data. However, next-generation sequencing (NGS) data are becoming more common, and often these data are sequenced at low to medium depth, where genotype calling can come with significant biases (Nielsen *et al.* 2012). This bias will be propagated into relatedness estimation and can lead to miss-classification of pairwise relatedness (Korneliussen and Moltke 2015). This issue can be avoided if instead of calling genotypes from low-depth data one accounts for the genotype uncertainty by summing over all possible unobserved genotypes and weighting each of these using genotype likelihoods (GLs). Several methods, like ngsRelate (Korneliussen and Moltke 2015; Hanghøj *et al.* 2019), and the very similar lcMLkin (Lipatov *et al.* 2015), have used this approach to estimate pairwise relatedness from homogeneous populations from low-depth sequencing data. Other methods that use external information also exist, such as SEEKIN (Dou *et al.* 2017), which uses imputation based on reference panels. However, none of these methods address relatedness estimates between individuals with admixed ancestry.

Here, we present a maximum likelihood method, NGSremix, that can estimate the relatedness coefficients (*R*) from low-depth sequencing data when individuals have admixed ancestry. The method takes GLs, admixture proportions, and ancestral allele frequencies as input. The GLs can be calculated using many software such as Samtools (Li *et al.* 2009) and ANGSD (Korneliussen *et al.* 2014), and ancestral allele frequencies can be estimated from clustering methods such a NGSadmix (Skotte *et al.* 2013) and PCAngsd (Meisner and Albrechtsen 2018) assuming that the ancestral populations are discrete and there is NGS or genotype data available from a sufficient number of individuals. The latter can be admixed individuals, unadmixed individuals, or both. We note that accurate ancestral allele frequencies estimated solely from admixed individuals usually require a large sample population and/or high levels of differentiation between the ancestral populations.

We also present a performance assessment of the method using both simulated data and real sequencing data from the 1000 genomes project and compare its performance to the commonly used state-of-the-art methods, PLINK (Purcell *et al.* 2007), KING (Manichaikul *et al.* 2010), relateAdmix (Moltke and Albrechtsen 2014), and ngsRelate (Hanghøj *et al.* 2019) when applied to admixed individuals with low-depth NGS data. Importantly, the assessment shows that NGSremix works well and clearly outperforms all the other methods when there is admixture and you have low-depth NGS data.

## Methods

### The model

The main objective of the model is to enable maximum likelihood estimation of the relatedness coefficients ($R=(k_{0},k_{1},k_{2})$) for two individuals, A and B, with ancestry from one or more of *K* different populations. We assume that we have NGS data from *M* variable diallelic sites for both individuals and denote this $X^{A}=(X_{1}^{A},X_{2}^{A},\ldots ,X_{M}^{A})$ and $X^{B}=(X_{1}^{B},X_{2}^{B},\ldots ,X_{M}^{B})$. Furthermore, we assume that the paired ancestry proportions for both individuals, denoted $\Phi^{A}=(\phi_{11}^{A},\phi_{12}^{A},\phi_{21}^{A},\ldots ,\phi_{KK}^{A})$ and $\Phi^{B}=(\phi_{11}^{B},\phi_{12}^{B},\phi_{21}^{B},\ldots ,\phi_{KK}^{B})$ are known. Finally, we assume that for each site the ancestral allele frequencies for the *K* populations, *F*, are known. The frequencies, *F*, can be defined in terms of either of the two alleles, but for sequence data the frequency is often defined in terms of the alternative (nonreference) allele. In practice, we estimate the ancestral allele frequencies *F* using NGSadmix (Skotte *et al.* 2013) and the paired ancestry proportion is estimated as described in Supplementary Material Section Estimation of paired ancestries and implemented in NGSremix. The paired ancestry proportions are simply the proportions of sites in the genome with a certain combination of ancestry, *i.e.*, $\phi_{a_{1},a_{2}}^{A}$ denotes the proportion of individual A’s genome where the first allele is from population $a_{1}$ and the second allele is from population $a_{2}$.

**Figure 1:**
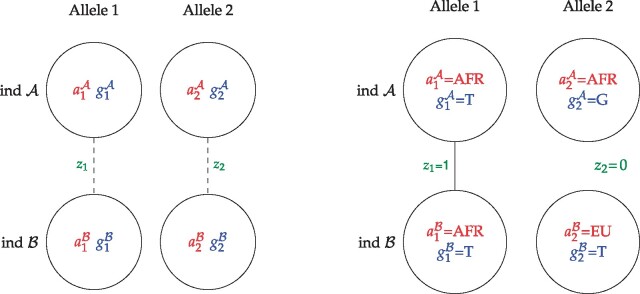
Left: Diagram of the unobserved state of the latent variables, *z*, *a*, $g^{A}$, $g^{B}$, in a site. The circles represent the two alleles of each of the two individuals A and B. Specifically, for each of these individuals, *e.g.*, individual A, the first allele has an unobserved ancestry state $a_{1}^{A}$, which can take any value among the *K* possible ancestral populations and an unobserved allelic state, $g_{1}^{A}$. Similarly, the second allele has an unobserved ancestry state, $a_{2}^{A}$, and an unobserved allelic state, $g_{2}^{A}$, where $\left(g_{1}^{A},g_{2}^{A}\right)$ constitutes the unobserved ordered genotype, $g^{A}$, for the individual. Lines indicate the unobserved IBD states, *i.e.*, whether the first alleles of the two individuals are IBD, $z_{1}$, and whether the second alleles of the two individuals are IBD, $z_{2}$. Right: Example diagram with a set of realized values for all the latent variables for two African American individuals with genotypes T/G and T/T ($g^{A}=(T,G)$ and $g^{B}=(T,T)$), who share their first allele IBD ($z_{1}=1$), which is possible because these alleles have the same African ancestry ($a_{1}^{A}=a_{1}^{B}=\mathrm{AFR}$) and the same allelic state ($g_{1}^{A}=g_{1}^{B}=T$). The second allele of the two individuals cannot be IBD $\left(z_{2}\right)$ because the two alleles originate from different ancestral populations ($a_{2}^{A}=\mathrm{AFR}\neq a_{2}^{B}=EU$) and/or because they have different allelic states $\left(g_{2}^{A}=G\neq g_{2}^{B}=T\right)$. Note that we here used T and G instead of 0 and 1 as possible allelic states and AFR and EU instead of 1 and 2 as possible ancestral states to make the example more concrete.

Using this terminology, we write up the likelihood function for *R*. Because we assume $\Phi^{A}$, $\Phi^{B}$ and *F* are known we have for simplicity not included them in this likelihood function. On the other hand, for each site the four alleles and their ancestry ($a=(a_{1}^{A},a_{2}^{A},a_{1}^{B},a_{2}^{B}$)), IBD status ($z=(z_{1},z_{2})$), and ordered genotypes ($g^{A}$ and $g^{B}$) are not observed, see Figure 1. Therefore, we include these as latent variables and weight each possible value of these by their probability. Note that even though we describe the model with ordered genotypes we do not require phased input data. Instead, we sum over all possible genotype orderings. The likelihood function can then be written a*s*

$$\begin{array}{c} P(X^{A},X^{B}|R)=\prod_{j=1}^{M}\sum_{z\in {\left\{0,1\right\}}^{2}}\sum_{a\in {\left\{1,..,K\right\}}^{4}}\sum_{g^{A},g^{B}\in {\left\{0,1\right\}}^{2}}P(X_{j}^{A}|G_{j}^{A}=g^{A})\\ \;\;\;\;\;\;\;\;\;\;\;\;\;\;\;\;\;\;\;\;\;\;\;\;P(X_{j}^{B}|G_{j}^{B}=g^{B})P(G_{j}^{A}=g^{A},G_{j}^{B}=g^{B}|A_{j}=a,Z_{j}=z)\\ \;\;\;\;\;\;\;\;\;\;\;\;\;\;\;\;\;\;\;\;\;\;\;P(A_{j}=a|Z_{j}=z)P(Z_{j}=z|R), \end{array}$$

where $z=(z_{1},z_{2})$ with $z_{1}$ indicating whether allele 1 of individual A and B are IBD and $z_{2}$ indicating whether the two individuals’ allele 2 are IBD. Furthermore, $a=(a_{1}^{A},a_{2}^{A},a_{1}^{B},a_{2}^{B})$ are the unobserved ancestral populations of the two individuals’ two alleles and $g^{A}$ and $g^{B}$ are the ordered genotypes for both individuals. Note that we sum over all possible *ordered* genotypes such that both alleles from individual 1 can be IBD with any of the two alleles of individual 2, if they are from the same ancestry and have the same allelic state. $P(X_{j}^{A}|G_{j}^{A}=g^{A})$ and $P(X_{j}^{B}|G_{j}^{B}=g^{B})$ are GLs that represent the information from the sequencing data, which can be calculated as described in Section 2.2.3 *e.g.*, using ANGSD (Korneliussen *et al.* 2014). The rest of the components of the likelihood function and a detailed description of how the likelihood function is derived can be found in Supplementary Material Section Derivation of the likelihood function.

To obtain maximum likelihood estimates of the paired ancestry Φ and the relatedness coefficients *R*, we are using Expectation-Maximization (EM) algorithms. The EM algorithms are described in the Supplementary Material Section EM algorithm and Estimation of paired ancestries. We defined convergence as the euclidean distance between the current and the previous parameter estimates being less than $10^{-6}$. For faster convergence, we implemented an accelerated EM algorithm using the squared iterative approach (S3) developed by Varadhan and Roland (2008). Convergence was reached on average after 20 iterations and no convergence issues were observed in the tested data sets. The computation time for NGSremix and the other methods used in this study is available in Supplementary Table S2. The method is implemented in C/C++ in a multi-threaded software and is available on Github https://github.com/KHanghoj/NGSremix.

### Simulation of data

Data were simulated to validate NGSremix and compare its performance with the existing methods: PLINK (Purcell *et al.* 2007), KING (Manichaikul *et al.* 2010), relateAdmix (Moltke and Albrechtsen 2014), and ngsRelate (Hanghøj *et al.* 2019). Using allele frequencies from Northern Europeans from Utah (CEU) and Yoruba in Ibadan, Nigeria (YRI) samples from the 1000 genomes project (Auton *et al.* 2015) we simulated NGS data with 100,000 diallelic sites for 10 pairs of individuals for each of 6 different relationship types: unrelated individuals (R=(1,0,0)), full siblings (R=(0.25,0.5,0.25), parent-offspring (R=(0,1,0)), half siblings (R=(0.5,0.5,0)), first cousins (R=(0.75,0.25,0)). The performance of the methods was evaluated using NGS data simulated based on 5 different average depths: 1x, 2x, 4x, 8x, and 16x. Data were generated by first simulating genotypes for each pair of individuals (see Simulation of genotypes). Next, the genotypes were used to simulate NGS data (see Simulation of NGS data) and finally GLs were calculated (Section Calculation of genotype likelihoods). Finally, we estimated ancestral allele frequencies from these GLs using NGSadmix (Skotte *et al.* 2013), that accounts for genotype uncertainty, allowing for two ancestral sources and used these estimates along with the GLs as input to our method.

As input to ngsRelate, we only used the GLs. For PLINK and KING that both require genotype data, we called genotypes from the calculated GLs by choosing the genotype with the highest GL and used these as input. For RelateAdmix that requires genotypes as well as admixture proportions and ancestral allele frequencies, we first used the called genotypes to estimate admixture proportions and ancestral allele frequencies using ADMIXTURE (Alexander *et al.* 2009) and then used these estimates as well as the called genotypes as input.

#### Simulation of genotypes

For unrelated pairs, we simulated genotypes without linkage disequilibrium by first randomly sampling an admixture proportion between the two ancestral populations (CEU and YRI) of 0, 0.25, 0.5, 0.75, or 1 for each individual. Then for each site, the ancestry of the alleles for each of the two individuals were sampled using the admixture proportions. Finally, the genotypes were sampled based on the sampled ancestral populations at that site and the allele frequencies from the 1000 genomes project (Auton *et al.* 2015). For the remaining relationship types, haplotypes were simulated by first simulating genotypes for an appropriate number of unrelated admixed founder individuals using the same approach as for the unrelated pairs. Next, we simulated data for the relevant pair by simulating offspring of these founders (and in some cases their offspring) using the relevant pedigree. For example, for siblings, we simulated data for two unrelated founder individuals (parents) and then simulated the siblings by simulating two offspring from these parents. In all cases, genotypes for an offspring of two parents were simulated by randomly sampling one allele from each parent at each site.

#### Simulation of NGS data

Based on the simulated genotypes, NGS data were generated for each individual as follows. First, the sequencing depth *d* at each site was sampled from a Poisson distribution with mean equal to the specified average sequencing depth. Next, *d* bases were sampled at each site *j*, $X_{j}=(b_{1},b_{2},\ldots ,b_{d})$, based on the individual’s genotype and a per base sequencing error probability, ϵ, of 0.005. In case of a sequencing error, the base is replaced with the other possible base at the site.

#### Calculation of genotype likelihoods

GLs were calculated using the model described by McKenna et al. (2010). However, for simplicity, we assume that there are only two alleles. At each site *j* and for each of the genotypes, $G_{j}=(g_{1},g_{2})$ where $g_{1},g_{2}\in \{0,1\}$, we calculated the likelihood of observing the NGS data $X_{j}=(b_{1},b_{2},\ldots ,b_{d})$, a*s*

$$\begin{array}{c} P(X_{j}|G_{j}=(g_{1},g_{2}))=P(X_{j}|G_{j}=(g_{2},g_{1}))\\ \;\;\;\;\;\;\;\;\;\;\;\;\;\;\;=\prod_{i=0}^{d}\left(\frac{1}{2}P(b_{i}|g_{1})+\frac{1}{2}P(b_{i}|g_{2})\right) \end{array}$$

where

$$P(b_{i}|g_{y})=\{\begin{array}{cc} \epsilon \;\; & b_{i}\neq g_{y}\\ 1-\epsilon \;\; & b_{i}=g_{y} \end{array}$$

Note that this model was originally described for unordered genotypes, but that the GLs for ordered genotypes are the same as for unordered genotypes. Thus, the method does not require phased information but only the GLs.

### Real data

We also tested NGSremix using real data from 417 individuals from the 1000 genomes project phase 3 release with 2504 individuals (Auton *et al.* 2015). These individuals have all been sequenced at low depth, however, they have also been genotyped using SNP chips and many of them have been sequenced with high depth exome and/or whole-genome sequencing. Therefore, we also have a high-quality genotype data set to compare the low-depth sequencing data to. Specifically, we focused on individuals from the admixed population of Americans of African Ancestry (ASW, *n* = 61). We also included European individuals (CEU, *n* =_**  **_99), African individuals (YRI, *n* =_**  **_108), and two populations representing a Native American component (PEL, *n* =_**  **_85; MXL, *n* =_**  **_64) in order to estimate accurate admixture proportions and ancestral allele frequencies for the admixed ASW individuals. To assess performance on low-depth NGS data, we analyzed the low-depth NGS data for these samples which have a median depth around 4x depth of coverage. First, we calculated GLs (-gl 2 -Q 30 -q 30) for all individuals using ANGSD (Korneliussen *et al.* 2014). When doing so we restricted the analysis to polymorphic sites in the high-quality genotype data for these five populations with a minor allele frequency of 0.05, followed by linkage disequilibrium pruning (PLINK1.90 –indep-pairwise 50 10 0.1) (Chang *et al.* 2015). A total of 270,428 sites were used for downstream analysis. Next, we estimated the ancestral allele frequencies using NGSadmix (Skotte *et al.* 2013), that accounts for genotype uncertainty, allowing for three ancestral sources. Finally, we applied NGSremix to the GL data, ancestry proportions, and ancestral allele frequencies to estimate the relatedness coefficients (R) for all pairs of individuals.

For comparisons using PLINK, KING and relateAdmix, we called genotypes from the low-depth NGS data by choosing the maximal GL for each site and each individual and used these as input. Furthermore, for relateAdmix we estimated ancestry proportions and ancestral allele frequencies from the called genotypes using ADMIXTURE (Alexander *et al.* 2009) and used these as additional input.

To validate the results obtained from the low-depth sequencing data, we performed a set of secondary analyses using the high-quality genotype data for the same individuals using the same set of genomic sites.

### Data availability

The 1000 genomes data used in this studies can be found here https://www.internationalgenome.org/data. Supplementary material is available at figshare: https://doi.org/10.25387/g3.14587164.

## Results

### Performance assessment using simulated data

**Figure 2:**
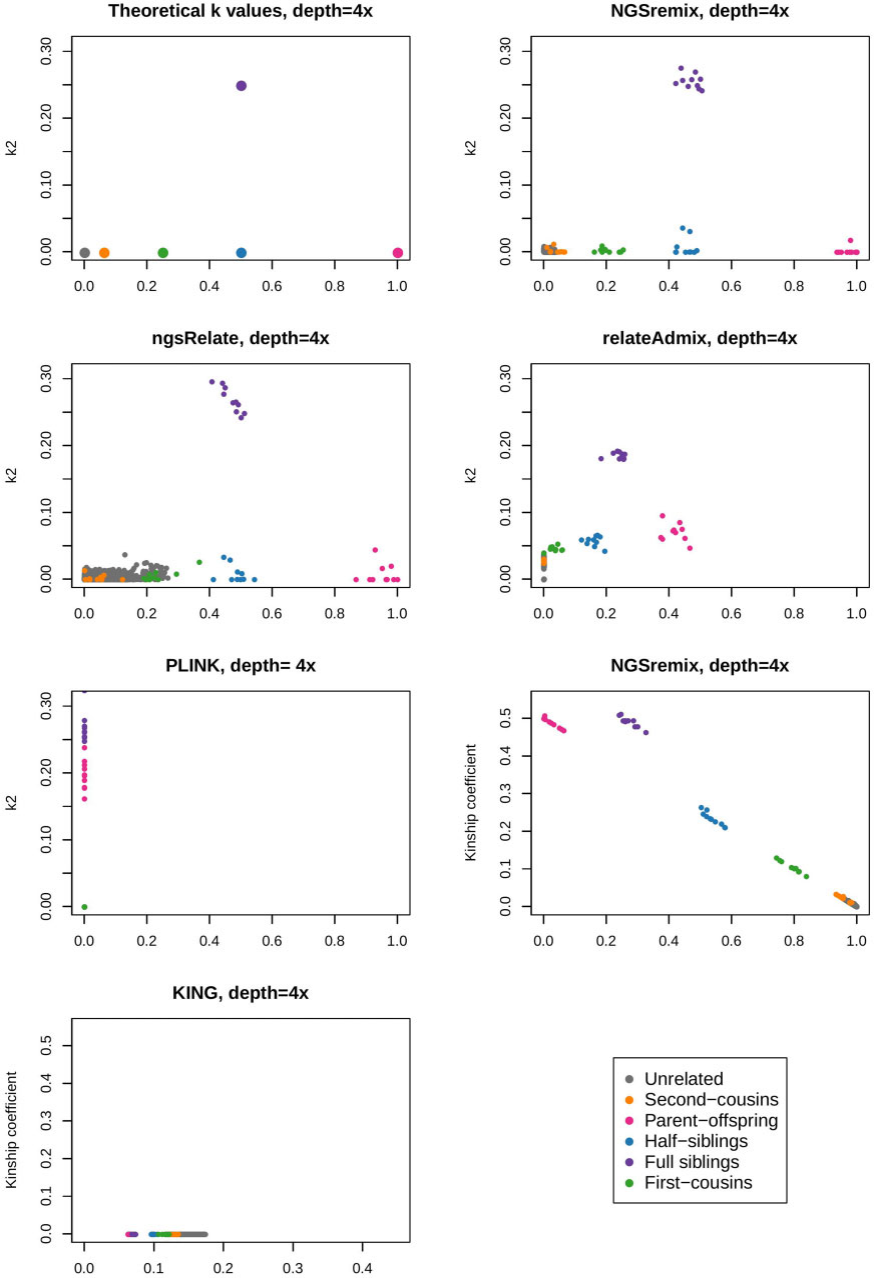
Estimated $k_{1}$ and $k_{2}$ or kinship for 120 simulated admixed individuals with 6 relationship types (10 of each type of related pairs) and an average depth of 4x. The first plot is the theoretical $k_{1}$ and $k_{2}$ values. Plots of the estimated $k_{1}$ and $k_{2}$ values are visualized for NGSremix, ngsRelate, reateAdmix, and PLINK. Plots of the kinship coefficient for NGSremix and KING are provided since KING only estimates kinship.

We first assessed the performance of NGSremix on simulated data. We simulated genotype data for 6 different relationship types and various admixture scenarios based on allele frequencies from the CEU and YRI 1000 Genomes population data. From these genotype datasets, we then simulated NGS data with depths 1x, 2x, 4x, 8x, and 16x. Finally, we estimated $k_{0}$, $k_{1}$, and $k_{2}$ values for the simulated NGS data for all pairs of individuals (*N* = 7140) using our NGSremix as well as the four commonly used state-of-the-art methods ngsRelate, relateAdmix, PLINK, and KING. Because KING estimates kinship and not *R*, the kinship coefficient for NGSremix and KING were also compared. When analyzing the simulated data with average depths of 8x or 16x, all methods can distinguish between full siblings, half-siblings, and parent-offspring (Supplementary Figures S1 and S2). However, only our NGSremix and relateAdmix obtain accurate estimates and allows clear separation of unrelated individuals from first-cousin. For simulated NGS data with an average depth of 4x, *i.e.*, low-depth NGS data, NGSremix is the only method that gives accurate results and allows the different relationship types to be distinguished from each other (Figure 2). ngsRelate give somewhat reasonable estimates for the first degree relationships, however, cannot separate the unrelated, second-cousin, and first-cousin. relateAdmix on the other hand, can for the most part distinguish between the relationships, but the relatedness coefficient estimates cannot be interpreted as IBD fractions making it difficult to use them *e.g.*, for relationship classification. The last two methods, PLINK and KING, performs worse in this scenario with estimated relatedness and kinship coefficients that are difficult to interpret. Similar results are obtained at lower depths (Supplementary Figures S3 and S4); even on 1x data our method performs fairly well, especially when increasing the number of sites, and is able to differentiate all relationship types except from unrelated and second cousins, see Supplementary Figure S6. As mentioned, NGSremix handles admixture by estimating paired ancestry proportions. We have also implemented a version of NGSremix without paired ancestry where we assume the ancestral populations to be discrete. The paired ancestry proportions result in less variance when the relatedness is high (Supplementary Figure S5).

### Performance assessment using real data

To further assess the performance of NGSremix we also applied the same methods to data from the admixed African American (ASW) individuals from the 1000 genomes project. We modeled the ancestry of ASW using a reference panel including populations with European (CEU), African (YRI), and Native American (represented by MXL and PEL) ancestries. First, we applied the methods to high-quality genotype data for these individuals to obtain estimates that are as close to the truth as possible, see Supplementary Figure S7.

NGSremix and relateAdmix that are both designed to take admixture into account, gave close to the same results and identified 5 parent-offspring pairs and one pair of second degree relatives (*i.e.*, a half sibling, avuncular, or grandparent-grandchild pair). These results are consistent with previous reports for these pairs (Coriell Institute, 2021). In contrast PLINK, ngsRelate and KING showed larger variance although they still manage to identify the same 5 parent-offspring pairs and the half-sibling pair. We note that two false positive third degree relatives (NA20274-NA20314 and NA20299-NA20314) are identified when modeling the ASW ancestry using only European and African populations in the reference panel, see Supplementary Figures S8 and S9. This is due to insufficient modeling of the high levels of Native American ancestry (40−65%) in these three individuals in a two-way admixture model (Supplementary Figure S8, Bottom) (Martin *et al.* 2017).

**Figure 3:**
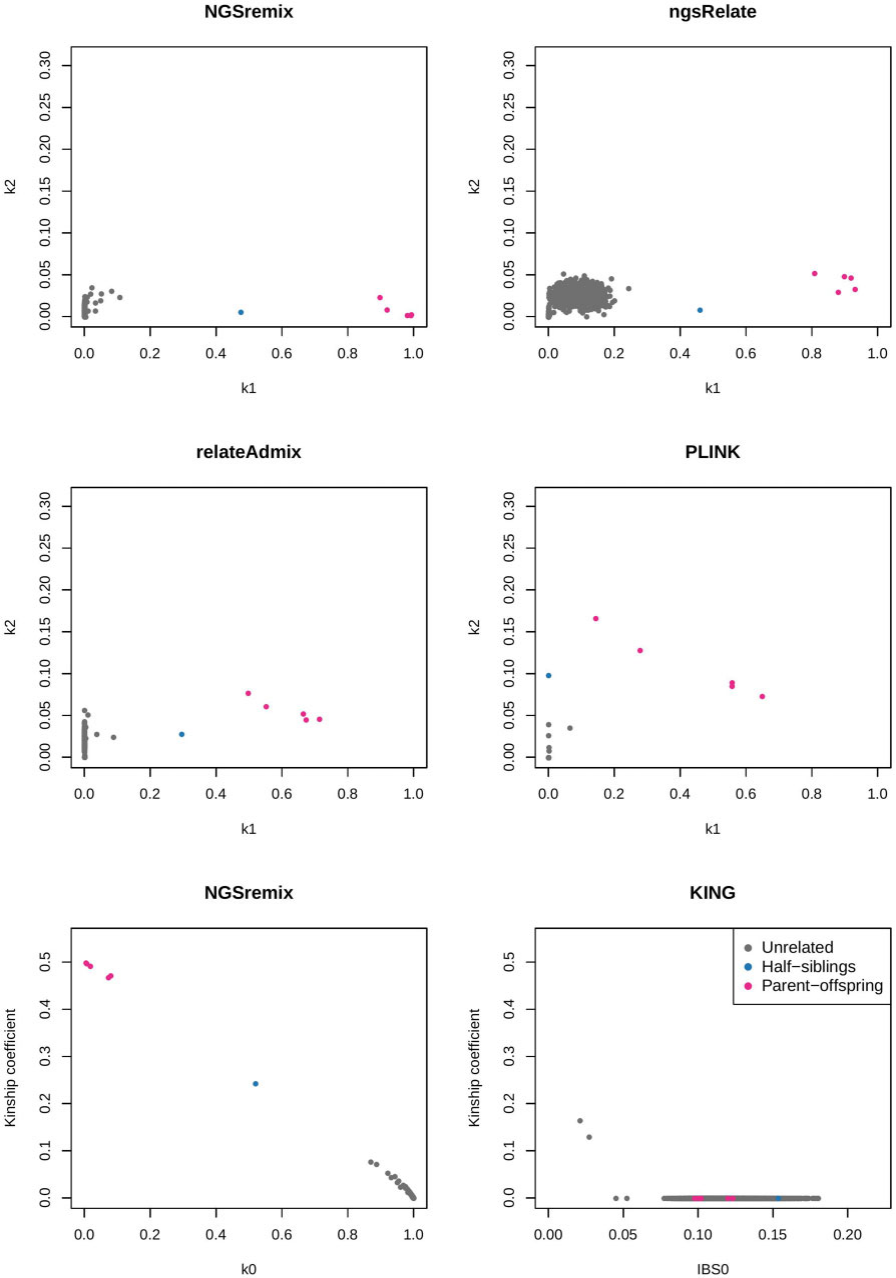
Inferred relatedness coefficients $k_{1}$ and $k_{2}$ or kinship, for 61 admixed African Americans from the 1000 genomes project sequenced at low depth. Plots of estimated *R* values are visualized for NGSremix, ngsRelate, relateAdmix, and PLINK. Plots of the kinship coefficient for NGSremix and KING are provided since KING estimates kinship and not *R*.

We next applied the methods to low-depth sequencing data (around 4x) from the same individuals. The results showed that our method is the only method that is able to identify the same related individuals as identified from the high-quality genotype data by both NGSremix and relateAdmix (Figure 3). ngsRelate have similar, suboptimal results as it did when it was applied to the high-quality genotype data, while relateAdmix, PLINK, and KING performed markedly worse. Hence, our method clearly outperforms the existing methods when applied to low-depth NGS data, not only for simulated data but also for real data. Also, we observed markedly worse results when applying NGSremix to genotypes called using a GL threshold than without using such a threshold. For this reason, we did not use a threshold when calling genotypes for the above-presented comparisons between NGSremix and the other methods (Supplementary Figure S10).

## Discussion

We have presented a new maximum likelihood-based software tool, NGSremix, for estimation of pairwise relatedness between admixed individuals sequenced at low depth. Using simulated data and real data from the 1000 genomes project, we have shown that our method is superior when used on admixed individuals sequenced at low depth compared to current state-of-the-art methods: PLINK, KING, relateAdmix, and ngsRelate. NGSremix’s accurate estimates of relatedness are due to the fact that it takes GLs as input instead of called genotypes, which is an advantage for low-depth NGS data, where genotypes cannot be called with high certainty. Second, NGSremix handles admixture by estimating paired ancestry proportions and including these in the model.

From the results of the simulations, we saw that the state-of-the-art methods that rely on genotypes as input could separate full-siblings, parent-offspring, and half-siblings when the average depth was 16x. However, KING, ngsRelate, and PLINK gave highly inaccurate relatedness/kinship estimates for the sibling and unrelated pairs. In contrast, relateAdmix (Moltke and Albrechtsen 2014) provided accurate estimates close to similar to the results of NGSremix, which is not a surprise as relateAdmix accounts for admixture and is methodically very similar to NGSremix. This shows that calling genotypes is a good option if the depth is high enough to call genotypes correctly and when an appropriate method that takes admixture into account is used. However, when lowering the simulated depth to 8x the genotype-based methods, including relateAdmix, obtained less accurate results compared to those based on 16x data. For depths below 8x, the uncertainty of genotype calls reached a level where the genotype-based methods began to give nonsensical results *e.g.*, for 4x data PLINK (Purcell *et al.* 2007) and KING (Manichaikul *et al.* 2010) estimate all $k_{1}$ coefficients and kinship coefficients to zero for all pairs of individuals regardless of relationship type. Thus, our results show that calling genotypes for low-depth NGS data can greatly bias estimation of pairwise relatedness. A common procedure to reduce this bias is to filter based on the genotype quality such that genotype calls with a low probability for being correct are disregarded. This might seem like a good way to remove the biases related to genotype uncertainty. However, because the probabilities assigned to heterozygous and homozygous calls will be very different with lower probabilities assigned to homozygous calls, these are more likely to be filtered away, which can result in further inflation of the genotype uncertainty biases (Supplementary Figure S10). This is not the case when using GLs, because they contain all information about the uncertainty of the genotype, while this information is lost when calling a genotype. Even for simulated data of depth 1x our method provided sensible results, however, albeit more noise was observed. We cannot make an exact statement about how low a depth our method can handle since it depends on number of SNPs, how admixed the individuals are, and the number of individuals. However, we expect it will not work well with lower depth since observing reads containing each individuals two alleles would be required at least at some sites.

All the tested methods in this study, including NGSremix, have a series of limitations. First, the estimated relatedness from the real data can deviate from the expectation for all relationship pairs except for parent-offspring and monozygotic twins, *e.g.*, the kinship coefficients for a pair of full-siblings is expected to be 0.5, but will often deviate from this exact value. This is due to biological variance during the recombination process, where a pair can share more/less IBD segments than expected (Thompson 2013) with variable variance depending on the relationship (Veller *et al.* 2020). This can in some cases make it challenging to disentangle the actual degree of a pair of relatives. Second, all the methods either assume allele frequencies are from discrete homogeneous populations or in the case of KING assumes that each pair of individuals is from the same homogeneous population. Third, for all of the methods except for KING the number of individuals included in the analysis is important since they are based on the assumption that allele frequencies can obtained. For NGSremix and relateAdmix the number of individuals is even more important because these methods are based on the assumption that the ancestral allele frequencies and admixture proportions can be accurately estimated using clustering methods such a NGSadmix (Skotte *et al.* 2013). The exact number of individuals per population needed in order to obtain accurate estimates from these methods will depend on the number of sites, the admixture patterns, and the degree of population differentiation. However, having less than 5 individuals representing a population will lead to inaccurate estimates (Jørsboe *et al.* 2017). Thus, NGSremix cannot be applied to studies were only a few individuals have been sequenced. Instead, it requires sequencing or genotype data for multiple individuals representing each ancestral population. Finally, NGSremix assumes that there is no inbreeding and that the markers are independent *i.e.*, no linkage disequilibrium. These assumptions are also shared with the other methods with the exception of ngsRelate which allows for arbitrary inbreeding patterns (Hanghøj *et al.* 2019). With regards to the assumption of no linkage disequilibrium then all of the methods can still be applied with data that has not been pruned. In this case, the models should be viewed as composite likelihood models which will have a different interpretation of the likelihood value. However, it will not affect the expected maximum likelihood point estimate (Lindsay 1988).

In conclusion, we have presented a maximum likelihood method for estimating relatedness for low-depth sequencing data that can be applied to admixed individuals. In simulations and real data from low-depth sequencing of admixed individuals the method outperforms other methods and gives reasonable results down to 1x sequencing.
